# Native Top‐Down Mass Spectrometry Reveals a Role for Interfacial Glycans on Therapeutic Cytokine and Hormone Assemblies

**DOI:** 10.1002/anie.202213170

**Published:** 2022-11-10

**Authors:** Di Wu, Carol V. Robinson

**Affiliations:** ^1^ Department of Chemistry University of Oxford Oxford OX1 3QZ UK; ^2^ Kavli Institute for Nanoscience Discovery University of Oxford Oxford OX1 3QU UK

**Keywords:** Biopharmaceuticals, Glycan, Glycoprotein, Native Mass Spectrometry, Oligomerization

## Abstract

Oligomerization and glycosylation modulate therapeutic glycoprotein stability and efficacy. The interplay between these two critical attributes on therapeutic glycoproteins, is however often hard to define. Here, we present a native top‐down mass spectrometry (MS) approach to assess the glycosylation status of therapeutic cytokine and hormone assemblies and relate interfacial glycan occupancy to complex stability. We found that interfacial O‐glycan stabilizes tumor necrosis factor‐α trimer. On the contrary, interferon‐β1a dimerization is independent of glycosylation. Moreover, we discovered a unique distribution of N‐glycans on the follicle‐stimulating hormone α subunit. We found that the interfacial N‐glycan, at Asn52 of the α subunit, interacts extensively with the β subunit to regulate the dimer assembly. Overall, we have exemplified a method to link glycosylation with assembly status, for cytokines and hormones, critical for informing optimal stability and bioavailability.

## Graphical abstract

Native top‐down mass spectrometry (MS) reveals subunit composition and glycan heterogeneity of therapeutic glycoprotein assemblies. By comparing the measured oligomer proteoforms with theoretical models, reconstituted from the dissociated subunits, we show that the interfacial glycan influences complex stability.

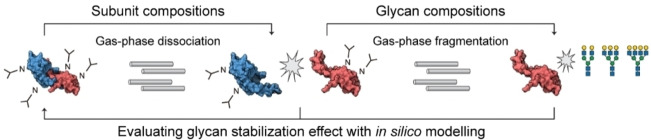

Oligomerization is a critical attribute of biopharmaceuticals in that it regulates both protein stability and efficacy. For monomeric biopharmaceuticals oligomerization, or aggregation, reduces the availability of the active form and increases the risk of immunogenicity. The oligomerization status of therapeutic protein complexes with multiple subunits by contrast is essential for maintaining their structural integrity and biological activity. Notably, most recombinant biopharmaceuticals produced from mammalian cell lines are glycosylated. Protein glycosylation modulates the biophysical properties of proteins by decreasing dynamics and stabilizing protein structures.^[1]^ Much effort has been made to customize protein glycosylation to improve therapeutic protein stability and efficacy.^[2]^ It remains however a significant challenge to address the roles of miscellaneous glycans, formed as products of non‐template driven glycosylation processes, on therapeutic glycoprotein assemblies by conventional approaches.^[3]^

Native mass spectrometry (MS), which involves maintaining and interrogating intact protein assemblies in the gas phase, is an emerging tool to probe the heterogeneity of intact glycoproteins and glycoprotein complexes.^[4]^ Recently, we developed a native MS approach to study the stabilization effects of interfacial lipids^[5]^ and a high‐resolution native top‐down MS platform to capture and interrogate heterogeneous membrane protein complexes.^[6]^ Here, we apply these native MS approaches to study the stabilization effects of interfacial glycans on therapeutic cytokine and hormone assemblies using a high‐resolution native MS platform.

Interferon‐β (IFN‐β) is an anti‐inflammatory cytokine involved in regulating immune responses. Recombinant human IFN‐β (IFN‐β1a) from Chinese hamster ovary (CHO) cells is a biopharmaceutical used to treat relapsing forms of multiple sclerosis (e.g., Avonex from Biogen and Rebif from EMD Serono). Human IFN‐β carries one core‐fucosylated N‐glycosylation site at Asn101^[7]^ (Figure 1A). A previous crystallographic study revealed that IFN‐β forms an asymmetric homodimer.^[8]^ One of the N‐glycans is known to be at the dimer interface. However, little is known about whether this N‐glycan stabilizes dimeric IFN‐β or regulates its formation. Here, we applied our native MS approach to analyze the assembly of IFN‐β1a sourced from European Pharmacopoeia Reference Standards.

**Figure 1:**
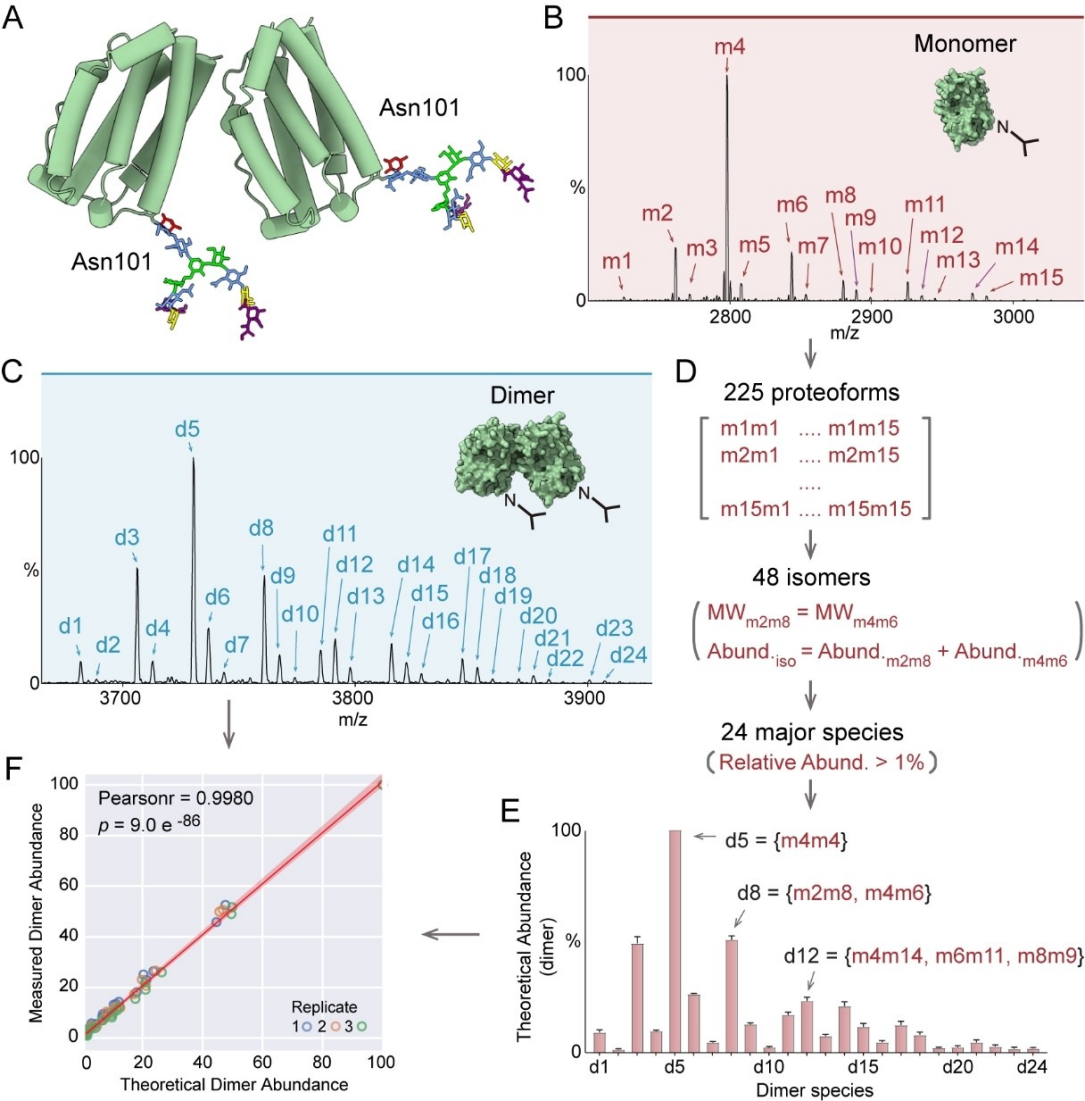
A) Structure of IFN‐β1a homodimer (PDB:1AU1). A bi‐antennary N‐glycan was modelled at α Asn101 of both subunits. The N‐glycan (on the left) is proximal to the interface of the asymmetric IFN‐β1a dimer. B) Native mass spectrum of IFN‐β1a monomer with charge state +8. Fifteen monomeric proteoforms (relative abundance >1%) are annotated (m1 to m15). C) Native mass spectrum of IFN‐β1a homodimer with charge state +12. Twenty‐four proteoforms (relative abundance >1%) are assigned (d1 to d24). D) Estimation of the relative abundance of IFN‐β1a dimer proteoforms. The theoretical proteoforms of IFN‐β1a dimers were calculated from a binomial expansion of the monomer intensities. 225 theoretical structural isoforms of IFN‐β1a dimers are binned to give 48 isomers. The abundances of the 24 major species were extracted and plotted as a bar graph in panel E. The theoretical compositions of d5, d8 and d12 are illustrated. F) Correlation of the theoretical and measured abundances of IFN‐β1a dimer proteoforms. Pearson correlation coefficient (Pearson r) is 0.9980.

We first recorded IFN‐β1a proteoforms of the monomers and homodimers using high‐resolution native MS (Figure 1B and C). We assigned 15 and 24 major proteoforms on IFN‐β1a monomer and dimers, respectively (all peaks with relative abundance >1% are considered). N‐glycosylation accounts for the major proteoforms (Supporting Information Figure 1). The spectrum of dimers exhibits a more complex pattern than the monomers, due to the combinations of each subunit with different proteoforms.

To examine the role of N‐glycans on IFN‐β1a dimerization, we first considered that dimerization of IFN‐β1a is independent of N‐glycosylation. Therefore, we calculated theoretical values, using a binomial model, for the relative abundances of dimer proteoforms based on the data of the monomer proteoforms (Figure 1D). As structural isomers with the same molecular weight cannot be distinguished by native MS we binned 225 theoretical proteoforms into 48 isomers and assigned 24 major species (with relative abundance >1%) (Figure 1E). We then plotted the theoretical abundances versus the measured values of the corresponding proteoforms to examine their correlation (Figure 1F). The Pearson correlation efficiency of these two datasets is 0.9980 (*p*=9 e^−86^). This correlation suggests a linear relationship between the population of the most abundant species from a theoretical prediction and the experimental measurement. This indicates that IFN‐β1a dimerization is independent of its N‐glycosylation status.

Next, we applied the native MS approach to another pro‐inflammatory cytokine, tumor necrosis factor‐α (TNF‐α). Human TNF‐α is a functional homotrimer with one interfacial O‐glycan at Ser80 on each subunit (Figure 2A). It is one of the most important cytokines regulating inflammatory responses, cell proliferation and apoptosis.^[9]^ There are several antibody‐based biopharmaceuticals and biosimilars that neutralize endogenous TNF‐α in autoimmune diseases, including the top‐selling Adalimumab (Humira), Etanercept (Enbrel) and Infliximab (Remicade). Recent studies have shown promising results using small molecules to disrupt the TNF‐α trimeric architecture to inhibit the endogenous TNF signaling pathway.^[10]^ Here, we investigated the structural implications of an interfacial O‐glycan on TNF‐α trimer formation and the disruption afforded by small molecule interactions.

**Figure 2:**
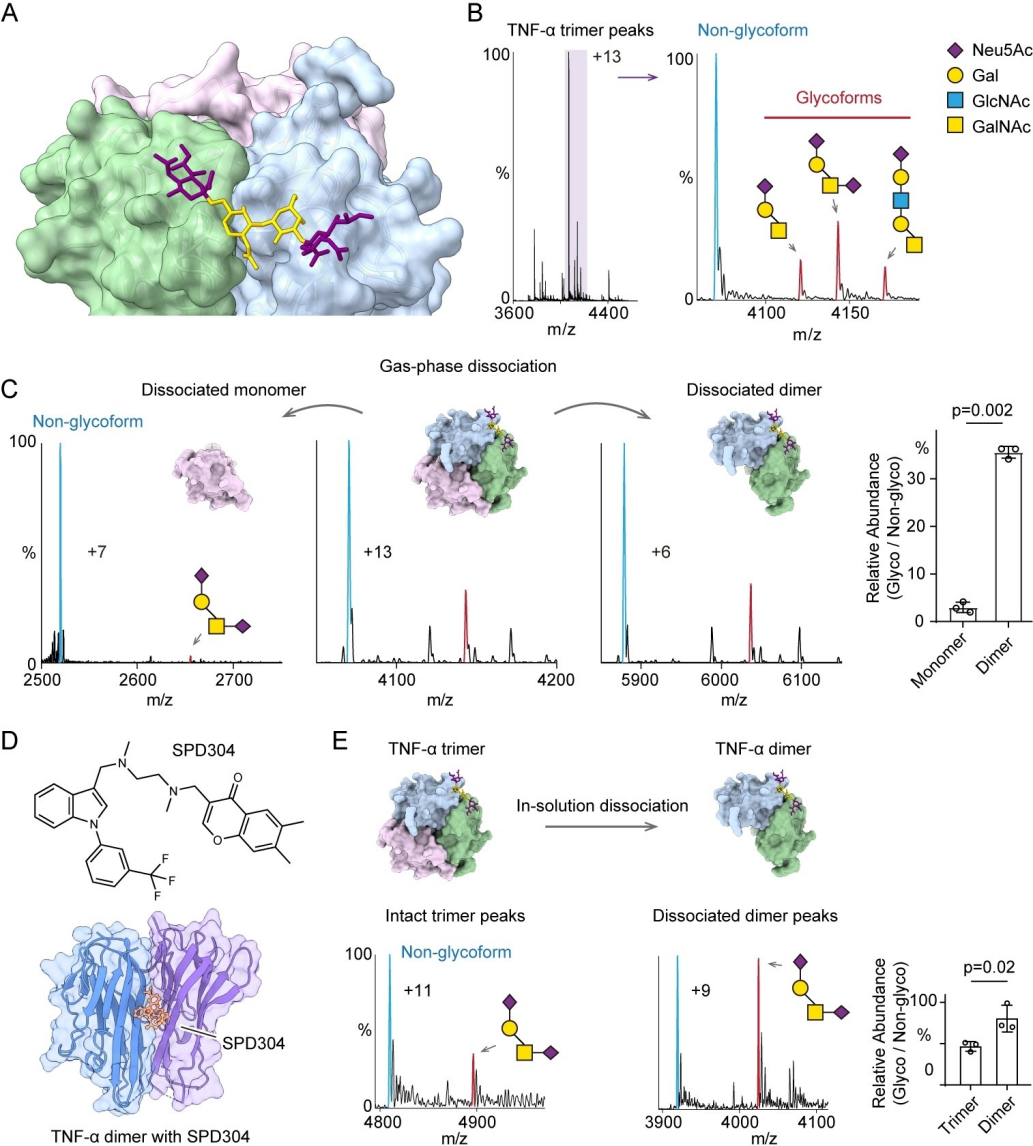
A) TNF‐α trimer with a tetra‐saccharide O‐glycan (disialyl‐T antigen, Neu5Acα1‐3Galβ1‐3(Neu5Acα1‐6)GalNAc) modeled on Ser80. B) Native mass spectrum of TNF‐α trimer with the peaks assigned to the non‐glycosylated form (blue) and the O‐glycosylated forms (red). The disialyl‐T antigen is the main species. C) Mass spectra of gas‐phase dissociated TNF‐α monomers and dimers. The O‐glycosylated subunit (disialyl‐T antigen, red peak) is principally in the dimer with very little glycosylated monomer ejected. The relative abundance of disialyl‐T antigen in dissociated monomers and dimers is plotted (bar graph). Bars show mean±standard deviation with data (dots) from three independent experiments. D) The structure of SPD304 and the crystal structure of TNF‐α dimer with SPD304 (PDB: 2AZ5). E) Native mass spectra of TNF‐α trimer following in‐solution disruption with 125 μM SPD304 in 1 % DMSO. The in‐solution dissociated dimers carry a higher level of disialyl‐T antigen. The bar graph shows the relative abundances of glycosylated trimer and dimer with disialyl‐T antigen. Bars show mean±standard deviation with data (dots) from three independent experiments.

First, we analyzed trimeric recombinant TNF‐α from human embryonic kidney 293 (HEK293) cells. We found that the trimer carries only one core 1 O‐glycan with different sialylation and extensions (Figure 2B). The disialyl‐T antigen (Neu5Acα1‐3Galβ1‐3(Neu5Acα1‐6)GalNAc, *N*‐acetylneuraminic acid, Neu5Ac; galactose, Gal; *N*‐acetylgalactosamine, GalNAc) is the most abundant O‐glycan modification on TNF‐α complexes. Hence, we focused on the analysis of this O‐glycan for the following native MS analysis. The TNF‐α monomer peaks are relatively low in native MS, so we cannot directly access the glycosylation status of the free monomers (Supporting Information Figure 2). We, therefore performed gas‐phase dissociation using higher‐energy collisional dissociation (HCD) to disrupt the TNF‐α trimers to monomer and dimer (Figure 2C). We found that the dissociated dimers carry a higher population of O‐glycan proteoforms compared to the dissociated monomers (Figure 2C). This observation implies that the O‐glycans stabilize the structure of the glycosylated subunit thereby resisting its unfolding and ejection by gas‐phase dissociation.

Next, we evaluated the O‐glycan stabilization effect by employing an in‐solution dissociation experiment. We incubated TNF‐α with SPD304, a small molecule that disrupts TNF‐α trimerization (Figure 2D).^[11]^ Then, we measured the relative abundance of the O‐glycosylated proteoform on the in‐solution dissociated dimers using native MS (Figure 2E). We found that the abundance of the O‐glycosylated dimer is increased relative to the untreated TNF‐α timer. This in‐solution dissociation experiment indicates that the O‐glycan stabilizes the TNF‐α dimeric subcomplexes. Together, the gas‐phase and in‐solution dissociation experiments suggest the interfacial O‐glycan plays a significant role in stabilizing the TNF‐α assembly.

Next, we extended this native MS approach by increasing the heterogeneity of the biopharmaceutical under investigation. The human follicle‐stimulating hormone (FSH) is a heterodimer with subunit α (glycoprotein hormone alpha chain) and subunit β (follitropin subunit beta) (Figure 3A). FSH carries four N‐glycans, two on each subunit. It is the key regulator in pubertal development and reproductive processes. Follitropin alpha is recombinant human FSH expressed in Chinese hamster ovary (CHO) cells.^[12]^ Currently, there are three follitropin alpha drugs marketed for fertility treatments including Gonal‐F, Follistim and Menopur. The extent of both α2‐6 sialylation and core‐fucosylation of FSH purified from CHO cells is less than that of FSH purified from urine .^[13]^ Here, we analyzed follitropin alpha from the European Pharmacopoeia Reference Standards using high‐resolution native MS. We removed sialic acid residues from follitropin alpha using α2‐3,6,8,9 neuraminidase A digestion to simplify the native top‐down MS characterization. The desialylation treatment is unlikely to affect FSH heterodimer conformations.^[14]^

**Figure 3:**
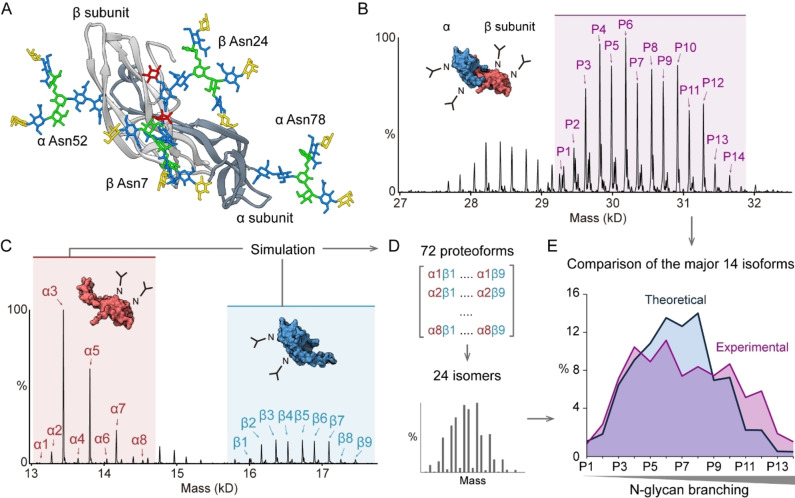
A) Structure of follitropin heterodimer. The α and β subunits are colored in dark and light grey, respectively. Four bi‐antennary N‐glycans were modelled at Asn52 and Asn78 in the α subunit, and Asn7 and Asn24 in the β subunit. B) Zero‐charged spectrum of desialylated follitropin heterodimer. The fully glycosylated forms are labelled (P1 to P14 peaks). Lower mass peaks correspond to heterodimers with 3N‐glycans. C) Zero‐charged spectrum of follitropin α and β subunits from denaturing MS analysis. The major peaks of α and β subunits were assigned with red (α1 to α8) and blue (β1 to β9) arrows, respectively. D) Estimation of follitropin dimer proteoforms. The 72 theoretical dimer proteoforms were calculated using the binomial model and binned to 24 isoforms. E) The comparison of theoretical and experimental follitropin dimer proteoforms (P1 to P14).

We found that the major species are fully glycosylated with four N‐glycans (Figure 3B and Supporting Information Figure 3), in line with a previous proteomics study.^[15]^ We annotated major proteoforms and revealed that N‐glycan branching accounts for the heterogeneity (Supporting Information Figure 3). To obtain proteoform information from each subunit, we dissociated the follitropin heterodimer in solution using 1 % formic acid and performed denaturing MS to analyze the α and β subunits separately (Figure 3C). We assigned the major proteoforms on each subunit (relative abundance >1%) and simulated the heterodimer proteoforms using the binomial model. The initial assumption in this model is that the assembly of the follitropin dimer is independent of the N‐glycan status of each subunit (Figure 3D). We then extracted 24 isomers and compared the 15 major forms with the corresponding observed values (Figure 3E). We found that the proteoforms with highly branched N‐glycans are more abundant than the theoretical prediction (P9 to P14 proteoforms). This result therefore implies that the assembly of the follitropin dimer is not independent of N‐glycan structures, but rather suggests that N‐glycan branching may regulate follitropin dimer assembly.

To explore how N‐glycan branching events, in terms of bi‐, tri,‐ and tetra‐antennary N‐glycans alter follitropin dimer assembly, we isolated single proteoforms of the follitropin heterodimers in the gas phase (P3 to P12 series). We performed gas‐phase dissociation of each proteoform in the P3 to P12 heterodimer series to examine the detailed α and β subunit heterogeneities (Figure 4A and Supporting Information Figure 4). We found the distributions of the α subunit are different between the predicted and experimental datasets (Figure 4B). The theoretical prediction using the binomial model indicates that the α3 subunit dominates in the P7 and P8 dimer proteoforms. However, the native MS data suggest that the α5 subunit is more abundant in these two dimer proteoforms (Figure 4C). The α5 subunit is 365 Da larger than the α3 subunit. Based on the monosaccharide residue masses we attributed this 365 Da mass difference to hexose (Hex, 162 Da) and *N*‐acetylhexosamine (HexNAc, 203 Da) residues. However, the α subunit carries two N‐glycosylation sites, namely αAsn7 and αAsn52. The Hex and HexNAc residues may be linked to one N‐glycan as an N‐glycan antenna (Hex‐HexNAc unit) or be distributed separately on the two Asn N‐glycan sites.

**Figure 4:**
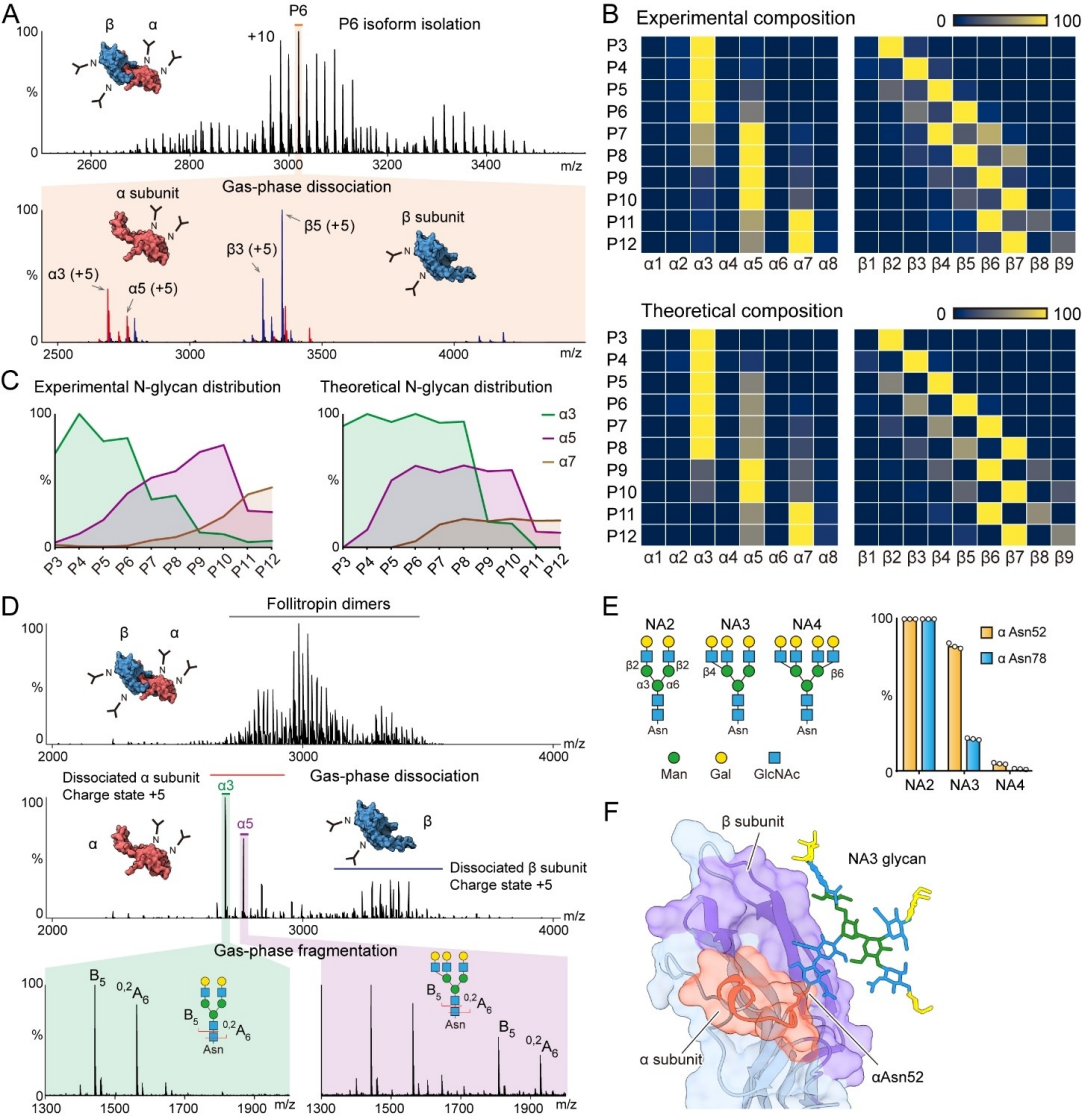
A) Native top‐down MS analysis reveals the detailed subunit proteoform composition of the follitropin dimer. The P6 proteoform of the dimer (P6 peak, *m*/*z* 3019, charge state +10) was isolated (with *m*/*z* 10 window) for gas‐phase dissociation using HCD (100 V). Each peak in the MS/MS spectrum (lower panel) was annotated to the corresponding subunits (α subunits—red peaks, β subunits—blue peaks). Using this approach, P3 to P12 proteoforms were further isolated and dissociated (Supplementary Figure 4). B) The experimentally measured and theoretical subunit compositions of follitropin dimers (P3 to P12) were plotted as heatmaps, respectively. C) The distribution of α3, α5 and α7 subunits in P3 to P12 dimers. D) Native top‐down native MS analysis revealed the N‐glycan composition of follitropin α subunits. The follitropin dimers were dissociated into α and β subunits using in‐source fragmentation of 100 V. The dissociated subunits α3 (*m*/*z*=2689, charge state +5) and α5 (*m*/*z*=2762, charge state +5) were selected with a window of *m*/*z* 10 and further fragmented using HCD of 25 V. The N‐glycan fragments in the low mass range were assigned to the corresponding structures. E) Structures of bi‐, tri‐ and tetra‐ antennary N‐glycans (NA2, NA3 and NA4) and their distributions at Asn52 and Asn78 in the α subunit. Bars show means with dots from three independent experiments. F) The structure of follitropin dimer with a tri‐antennary N‐glycan on α Asn52. The amino acids in the α and β subunits that contact the NA3 glycan in the 150 ns MD simulation trajectory are highlighted in orange and purple, respectively.

To examine the linkage information of these two monosaccharide residues we performed a native multi‐stage top‐down experiment (MS/MS/MS) to investigate the N‐glycan composition of the α3 and α5 subunits using an Orbitrap Eclipse mass spectrometer.^[6]^ Briefly, we dissociated the follitropin dimers to α and β subunits using in‐source fragmentation. We then selected and fragmented α3 and α5 subunits, respectively. We observed the B and ^0,2^A cross‐ring fragment ions of N‐glycans in the low mass range. The results showed that the subunit α3 carries two bi‐antennary N‐glycans while the subunit α5 carries one bi‐antennary and one tri‐antennary N‐glycan (Figure 4D and Supporting Information Figure 5). Further bottom‐up glycoproteomics analysis confirmed that the tri‐antennary N‐glycan is present primarily at Asn52 in the α subunit (αAsn52) (Figure 4E). Therefore, we attributed the regulation of follitropin assembly to the N‐glycan at Asn52 in the α subunit.

Moreover, we modeled the follitropin heterodimer with tri‐antennary N‐glycans and performed molecular dynamics (MD) simulations to probe the contacts between the N‐glycan at αAsn52 and the polypeptide chain of the β subunit. The MD simulation showed that the N‐glycan at αAsn52 makes extensive contacts with the β subunit (Figure 4F); the hydrogen bonding between the N‐glycan at αAsn52 and the β subunit likely contributes to the stabilization of the follitropin heterodimer (Supporting Information Figure 6). Further biophysical analysis of the highly branched follitropin with tri‐antennary N‐glycan on αAsn52 would help to unveil the structural basis of N‐glycan stabilization effects on follitropin.

In summary, we applied native top‐down MS to interrogate the role of interfacial glycans on therapeutic cytokine and hormone assemblies. We first applied our approach to IFNβ1a, and found that its dimerization is independent of its N‐glycosylation status. We further established that the interfacial O‐glycan on TNFα stabilizes the homotrimer architecture. Moreover, we revealed the interfacial N‐glycan at Asn52 in the follitropin α subunit regulates the assembly of the follitropin heterodimer. Together these results demonstrate not only a panoramic view of the glycan repertoire of therapeutical cytokine and hormone assemblies,[^16^, ^17^] but also the stabilization effect of glycans on intact biopharmaceutical complexes—a critical factor in activity and the long‐term storage of therapeutic proteins.

## Conflict of interest

The authors declare no conflict of interest.

## Supporting information

As a service to our authors and readers, this journal provides supporting information supplied by the authors. Such materials are peer reviewed and may be re‐organized for online delivery, but are not copy‐edited or typeset. Technical support issues arising from supporting information (other than missing files) should be addressed to the authors.

Supporting Information

## Data Availability

The raw mass spectra have been deposited on figshare (https://doi.org/10.6084/m9.figshare.11189660.v1).
