# Supplementary material for: Native Top‐Down Mass Spectrometry Reveals a Role for Interfacial Glycans on Therapeutic Cytokine and Hormone Assemblies
Source: Angew Chem Int Ed Engl. 2022 Nov 10;61(49):e202213170. doi: 10.1002/anie.202213170 (PMC10100379; doi:10.1002/anie.202213170)
Supplement: Supplementary file 1 — Supporting Information [file ANIE-61-0-s001.pdf]

Angewandte Chemie
Eine Zeitschrift der Gesellschaft Deutscher Chemiker

Supporting Information

Native Top-Down Mass Spectrometry Reveals a Role for Interfacial Glycans on Therapeutic Cytokine and Hormone Assemblies

D. Wu, C. V. Robinson**

Supplementary Information

This PDF file includes:

Methods
Supplementary Figures

Methods

Materials. IFNβ-1a (Y0001101) and follitropin (Y0001629) are European Pharmacopoeia Reference Standards from Merck. TNF-α, ammonium acetate (7.5 M solution), formic acid, dimethyl sulfoxide (DMSO) and SPD304 were purchased from Sigma-Aldrich. Neuraminidase (α2-3,6,8,9) were from New England Biolabs.

Native MS analysis. Glycoproteins were desalted with 1 M ammonium acetate using a Zeba spin desalting column (Thermo Scientific) and then diluted to 200 mM ammonium acetate for native MS analysis. The desalted glycoproteins were then loaded to in-house prepared gold-coated needles and analysed using an Orbitrap UHMR mass spectrometer (Thermo Fisher Scientific). The typical MS settings were spray voltage of 1.2 kV, source fragmentation of 50 V, source temperature of 150 °C, HCD collision energy of 0 V and resolution of 17500 at *m/z* 200. Trapping gas pressure was maintained at 6.5 for all measurements. The native mass spectra were processed using Xcalibur 4.1.

Proteomics analysis. Glycoproteins were buffer-exchanged to 100 mM Tris buffer (pH 8.0) containing 8 M urea and 5 mM dithiothreitol (DTT) then incubated at 56 ºC for 20 min and buffer-exchanged to 100 mM Tris buffer (pH 8.0) with 20 mM iodoacetamide (IAA) using an Amicon Ultra-0.5 centrifugal filter (10 kDa, MWCO, Millipore). The samples were then alkylated at room temperature for 20 min in the dark and buffer-exchanged to 50 mM NH_4HCO_3 (pH 8.0). The glycoproteins were then transferred into a new Eppendorf tube and digested with trypsin at 37 ºC overnight. The digested peptides were dried and reconstituted with 1% formic acid for LC-MS/MS analysis. The tryptic peptides (100 ng) were analysed on a Dionex Ultimate 3000 UHPLC coupled to an Orbitrap Eclipse Tribrid mass spectrometer (Thermo Fisher Scientific). The peptides were firstly loaded onto a 75 μm×2 cm pre-column and separated on a 75 μm×15 cm Pepmap C18 analytical column (Thermo Fisher Scientific) with a binary buffer system. Buffer A was 0.1% formic acid (FA) in 100% H_2O and buffer B was 0.1% FA in 80% acetonitrile with 20% H_2O. The Eclipse mass spectrometer was operated in data-dependant acquisition mode with one full MS scan followed by MS/MS scans with HCD fragmentation.

Denaturing MS analysis. The glycoproteins were diluted to 1% formic acid for denaturing. The denatured glycoproteins were then analysed on a Dionex Ultimate 3000 UHPLC coupled to an Orbitrap XL mass spectrometer (Thermo Fisher Scientific). The glycoproteins were directly loaded onto a 75 μm×15 cm Pepmap C8 analytical column (Thermo Fisher Scientific) with a binary buffer system. Buffer A was 0.1% formic acid (FA) in 100% H_2O and buffer B was 0.1% FA in 80% acetonitrile with 20% H_2O. The Orbitrap XL mass spectrometer was operated in MS scan mode. The denaturing mass spectra were analysed using UniDec software.

Native top-down MS analysis. The desialylated FSH dimer was analysed on an Orbitrap Eclipse Tribrid mass spectrometer (Thermo Fisher Scientific) [1]. The typical MS settings were spray voltage of 1.2 kV, source temperature of 150 °C. The follitropin heterodimers were dissociated with 100 V using in-source fragmentation. The dissociated α subunit peaks were selected using ion trap with a window of m/z 10. The selected ions were then injected into the ion routing multipole for further fragmentation.

The fragment ions were detected in the Orbitrap. The top-down mass spectra were analysed manually for N-glycan assignments.

Native MS data analysis. The native mass spectra were deconvoluted using UniDec software [2]. The theoretical molecular weights of IFN-β1a, TNF-α and FSH were calculated using amino acid and monosaccharide residue masses. The quantification of each proteoform was manually performed using Xcalibur.

Proteomics data analysis. The LC-MS/MS data were processed with PGlyco (version 2.0) [3] for glycopeptide identification. Quantification of the site-specific microheterogeneity was performed manually using Xcalibur (version 4.1, Thermo Fisher Scientific). The extracted ion chromatogram (XIC) of each glycopeptide was processed with 50 ppm mass tolerance and a 7-point Gaussian smoothing. The area under the curve (AUC) was integrated for glycopeptide quantification.

Denaturing MS data analysis. The mass spectra of denatured glycoprotein subunits were retrieved from LC-MS raw data using Xcalibur, and then deconvoluted using UniDec software.

Prediction of glycoprotein dimer proteoforms. The theoretical glycoprotein dimer proteoforms were calculated based on the hypothesis that the glycoprotein dimerization is independent of the glycosylation status of the monomer. The mass of the dimer proteoform D (M_D) can be calculated as:

$$M_D = M_i + M_j$$

whereas M_i and M_j are the masses of the monomer proteoforms i and j.

The corresponding relative abundance of the dimer proteoform D (A_D) can be calculated as:

$$A_D = A_i \times A_j$$

whereas A_i and A_j are the normalized relative abundances of the monomer proteoforms i and j.

The data were processed and plotted with seaborn library in Python 3.8.5. The Pearson correlation efficiency between the predicted and native MS measured datasets was calculated using pearsonr function in SciPy library.

Protein structure modelling. Protein structures of IFN-β1a dimer (PDB: 1AU1), TNF-α trimer (PDB: 1TNF), TNF-α dimer with SPD304 (PDB: 2AZ5) and FSH dimer (PDB: 1XWD) were retrieved from the PDB. The missing N-terminal sequence of the TNF-α trimer was patched using the full-length TNF-α structure from AlphaFold protein structural database (alphafold.ebi.ac.uk). A bi-antennary N-glycan were modelled to Asn101 in each IFN-β1a subunit using Glycan Reader and Modeler [4]. A disialyl-T antigen (Neu5Acα1-3Galβ1-3(Neu5Acα1-6)GalNAc) was modelled to Ser80 of TNF-α using Glycan Reader and Modeler. The protein structures were processed using University of California, San Francisco Chimera program (version X 1.2.5) [5].

Molecular dynamics simulation. The human FSH heterodimer structure was extracted from the crystal structure of FSH-FSHR complex (PDB: 1XWD) and used as a template for glycoprotein modelling. Tri-antennary N-glycans were added to Asn52 and Asn78 in subunit α, and Asn8 and Asn25 in subunit β using CHARM-GUI (http://www.charm-gui.org) [6,7]. The protein N-terminus and C-terminus were

patched with acetylation and methylamidation, respectively. The glycoprotein was then placed in a periodic box of TIP3P water molecules with 150 mM KCl. The box boundaries are 15 Å away from the glycoprotein. The CHARMM36m force field was used for the polypeptide chain and carbohydrate residues. All simulations were performed at 303.15 K. After 5000 steps of energy minimization, all atoms were equilibrated for 200 ps under constant particle number, volume and temperature (NVT) conditions. The simulations were then performed using under constant particle number, pressure and temperature (NPT) conditions using GROMACS (version 2018) [8]. The temperature was maintained at 303.15 K using a Nose-Hoover thermostat with a time constant of 1 ps. A Parrinello-Raham barostat was employed for pressure regulation. Van der Waals interactions were treated using a forced-based switching function between 10 and 12Å. Long-range electrostatics were treated with the particle-mesh Ewald (PME) method. SHAKE was used to constrain all bonds involving hydrogen atoms. The data analysis (RMSD, RMSF, native contacts, and hydrogen bonding) was performed using built-in functions of GROMACS. The protein structures were visualized using UCSF Chimera program.

Reference

1. Gault, J. *et al.* Combining native and 'omics' mass spectrometry to identify endogenous ligands bound to membrane proteins. *Nat. Methods* **17**, 505–508 (2020).
2. Marty, M. T. *et al.* Bayesian deconvolution of mass and ion mobility spectra: from binary interactions to polydisperse ensembles. *Anal. Chem.* **87**, 4370–4376 (2015).
3. Liu, M. Q. *et al.* PGlyco 2.0 enables precision N-glycoproteomics with comprehensive quality control and one-step mass spectrometry for intact glycopeptide identification. *Nat. Commun.* **8**, 1–14 (2017).
4. Park, S. J. *et al.* CHARMM-GUI Glycan Modeler for modeling and simulation of carbohydrates and glycoconjugates. *Glycobiology* **29**, 320–331 (2019).
5. Pettersen, E. F. *et al.* UCSF Chimera--a visualization system for exploratory research and analysis. *J. Comput. Chem.* **25**, 1605–1612 (2004).
6. Jo, S., Kim, T., Iyer, V. G. & Im, W. CHARMM-GUI: A web-based graphical user interface for CHARMM. *J. Comput. Chem.* **29**, 1859–1865 (2008).
7. Lee, J. *et al.* CHARMM-GUI Input Generator for NAMD, GROMACS, AMBER, OpenMM, and CHARMM/OpenMM Simulations Using the CHARMM36 Additive Force Field. *J. Chem. Theory Comput.* **12**, 405–413 (2016).
8. Abraham, M. J. *et al.* GROMACS: High performance molecular simulations through multi-level parallelism from laptops to supercomputers. *SoftwareX* **1**–**2**, 19–25 (2015).
9. Jurrus, E. *et al.* Improvements to the APBS biomolecular solvation software suite. *Protein Sci.* **27**, 112–128 (2018).

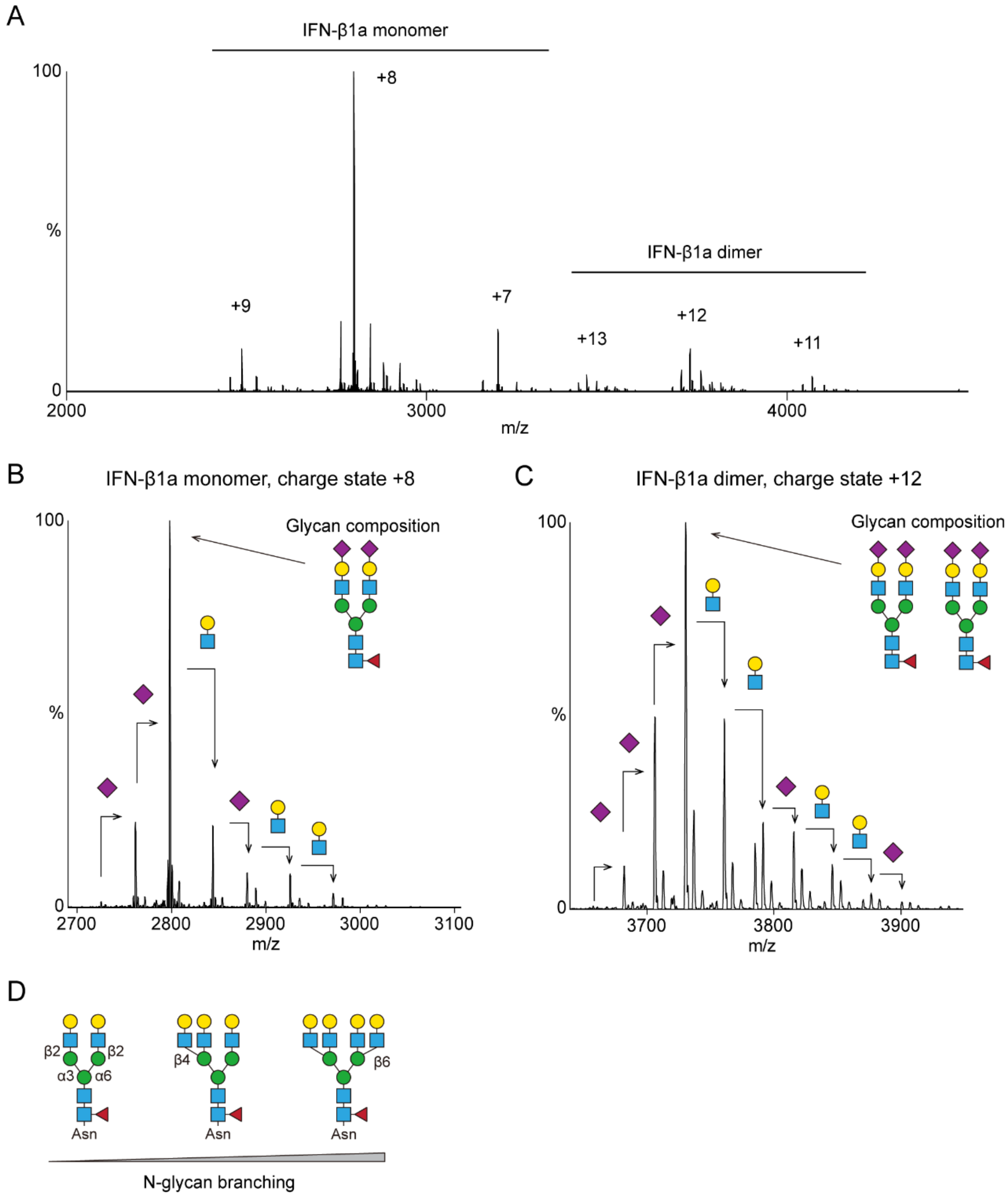

Supplementary Figure 1. A) Native mass spectrum of IFN-β1a in 200 mM ammonium acetate. The monomer and dimer peaks are labelled with the corresponding charge states. B) Annotation of the peaks of IFN-β1a monomer. C) Annotation of the peaks of IFN-β1α dimer. D) Illustration of N-glycan branching.

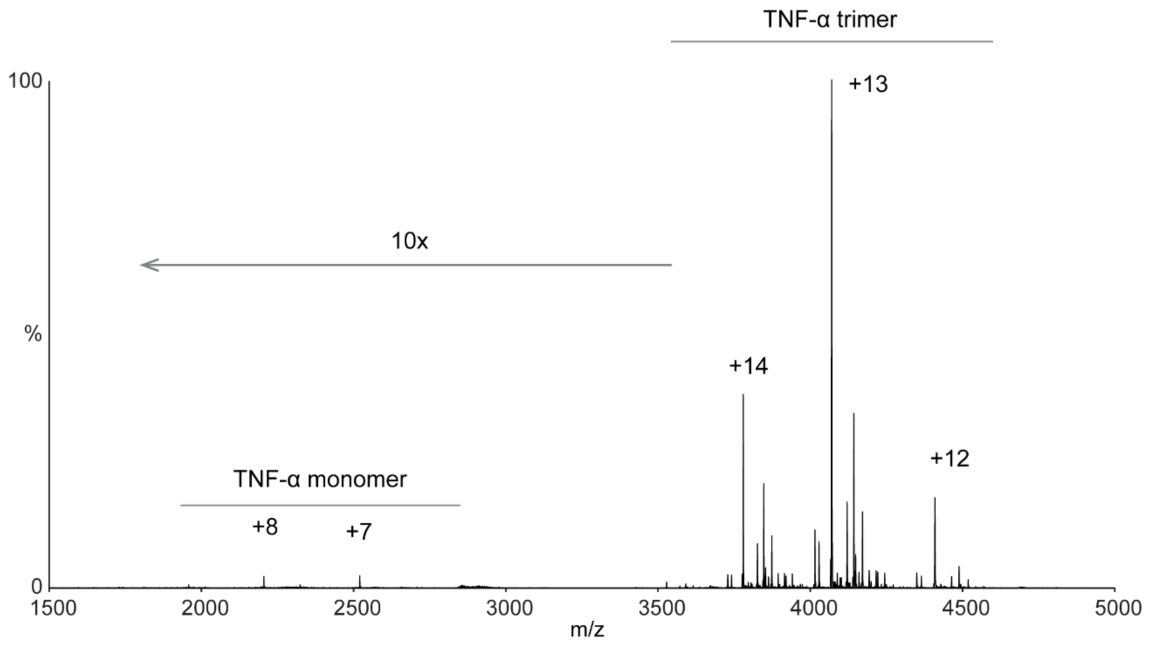

Supplementary Figure 2. Native mass spectrum of TNF-α trimer in 200 mM ammonium acetate. The relative intensities of the monomer peaks are less than 1% with respect to the trimer peaks.

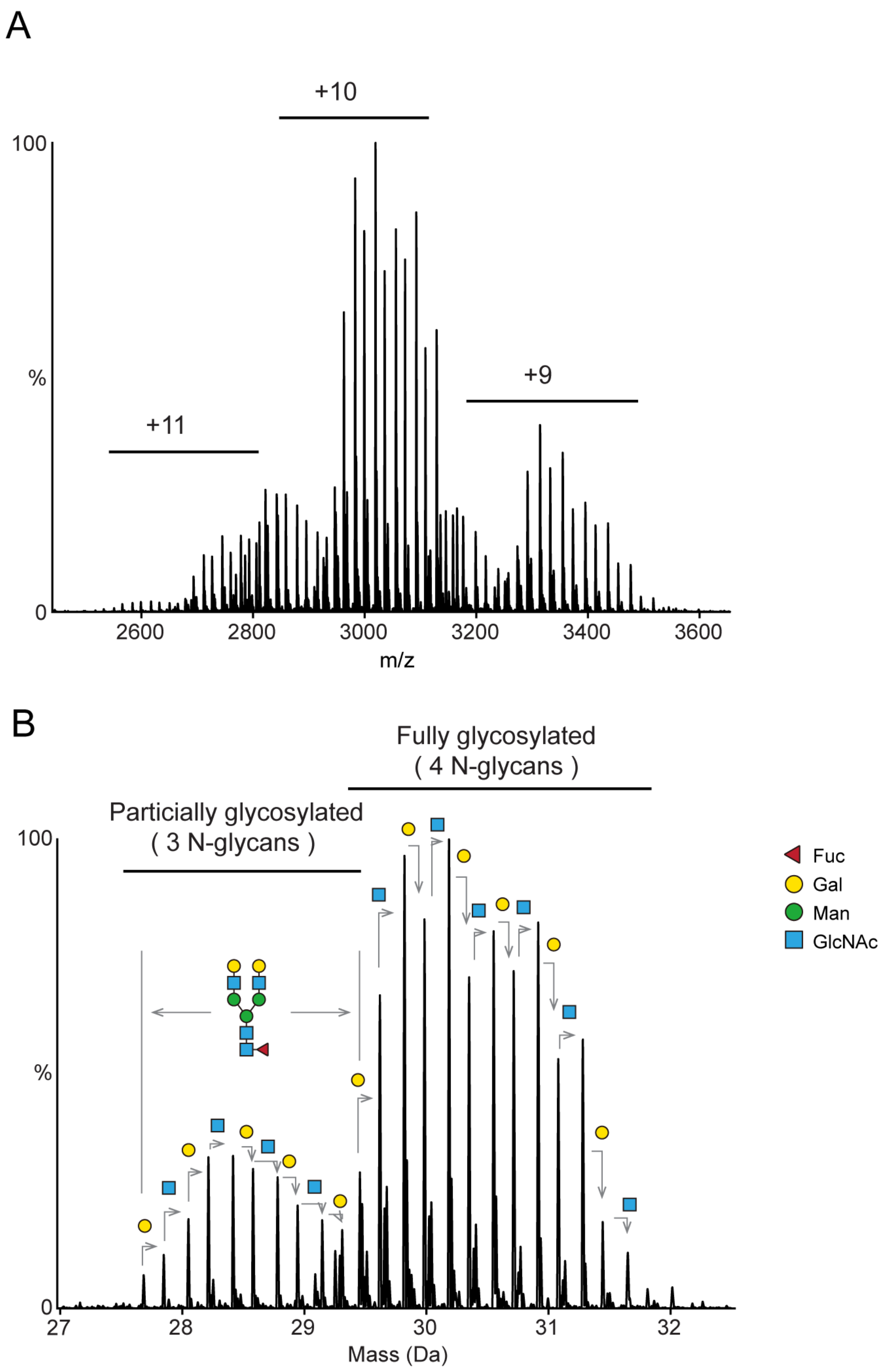

Supplementary Figure 3. A) Native mass spectrum of desialylated follitropin dimers. The peaks are labelled with the corresponding charge states. B) Zero-charged spectrum of desialylated follitropin dimer. The partially glycosylated and fully glycosylated peaks are annotated.

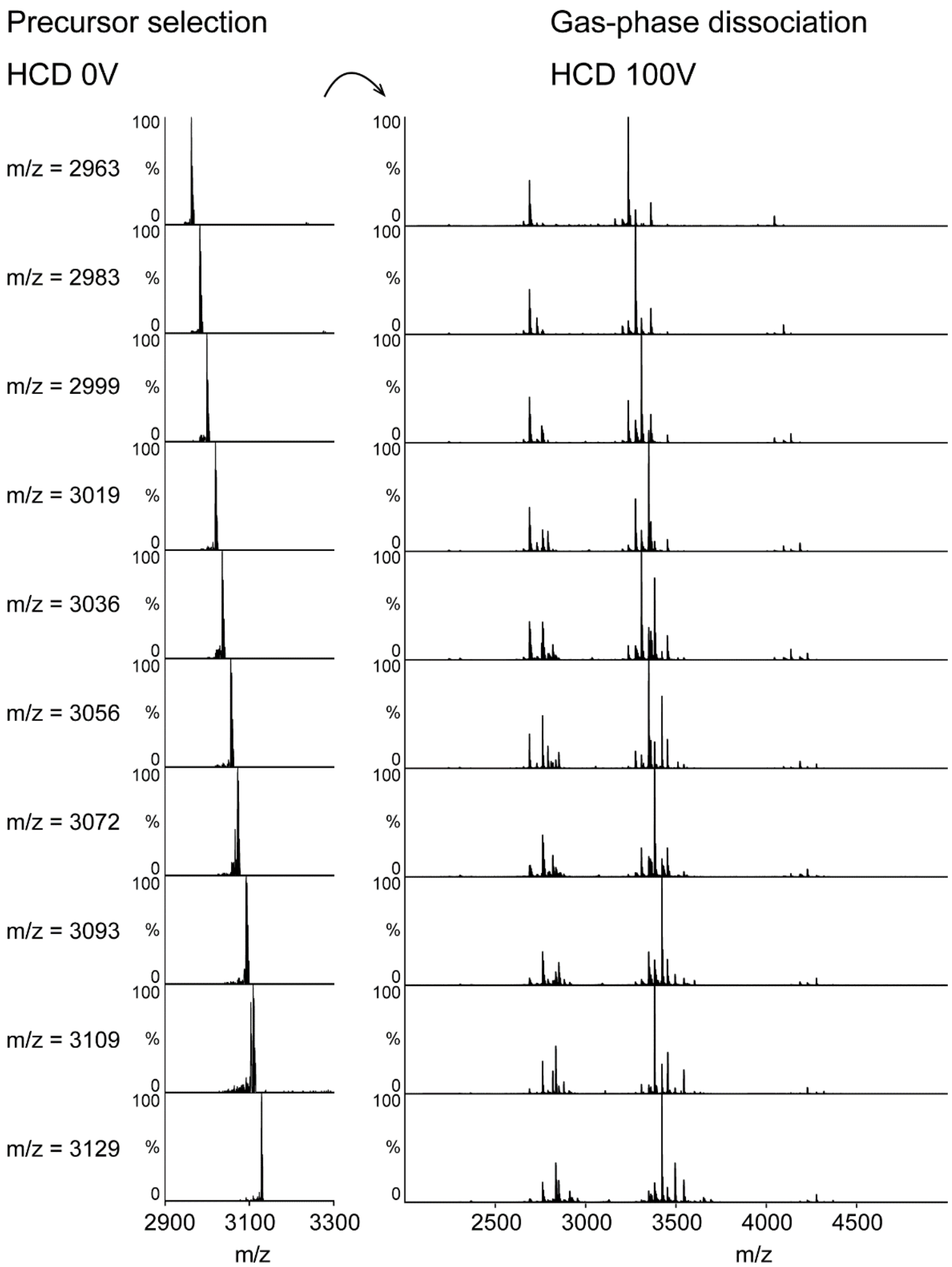

Supplementary Figure 4. Native top-down MS analysis of follitropin dimers. Charge state +10 dimer peaks (from m/z 2963 to 3129) are selected and dissociated into α and β subunits using higher-energy collisional dissociation (HCD) of 100V.

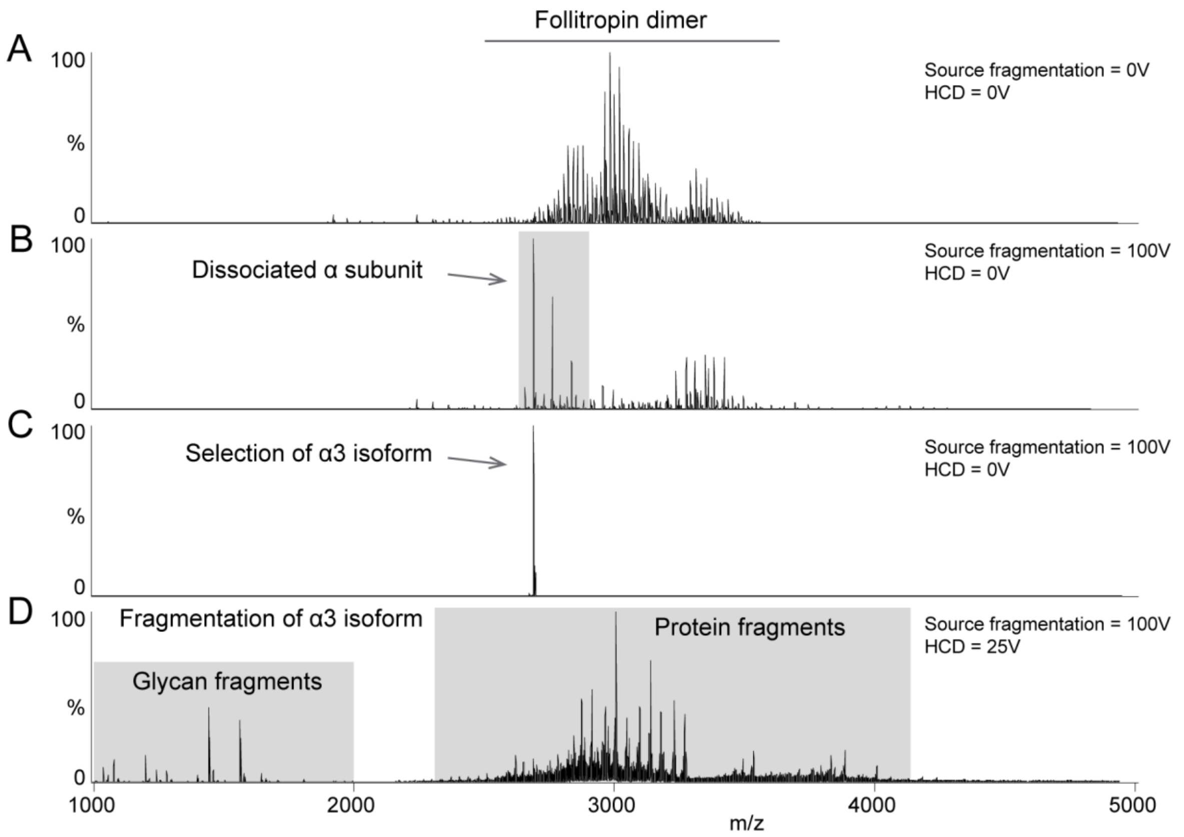

Supplementary Figure 5. Native top-down MS analysis of the glycan composition on α subunit. We firstly dissociated follitropin dimers (panel A) into α and β subunits using source fragmentation of 100V (panel B). Then, we selected the α3 subunit (charge state +5) with a window of m/z 10 (panel C) and further fragmented with HCD of 25V (panel D). The N-glycan fragments and protein fragments are highlighted, respectively.

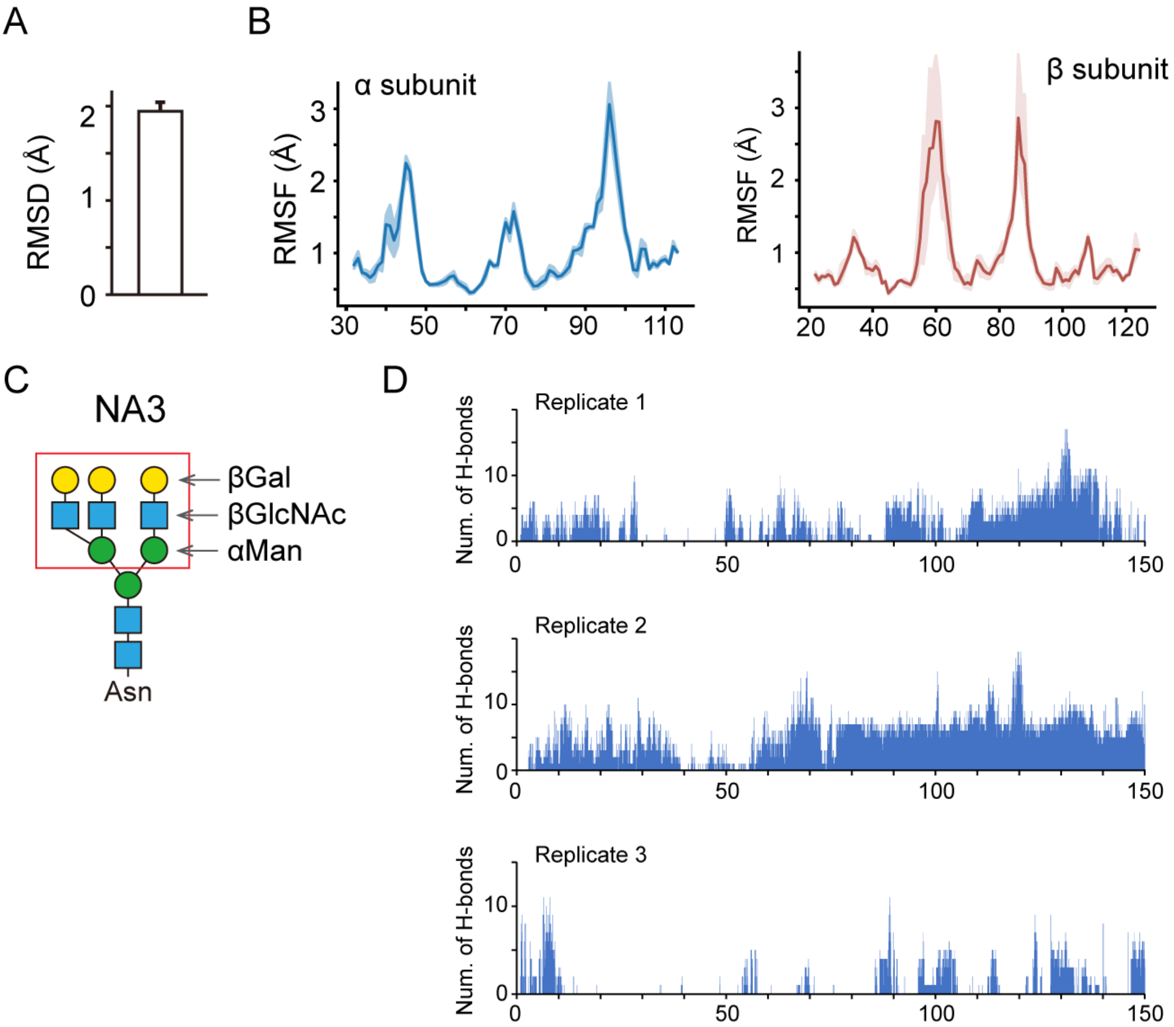

Supplementary Figure 6. A) The Cα atom root-mean-square deviation (RMSD) of glycosylated FSH in 150 ns MD simulations. The bargraph shows the mean ± standard deviation from three replicates. B) The Cα atom root-mean-square fluctuation (RMSF) plot of the α subunit and β subunit in 150 ns MD simulations. The 95% confidence intervals (three replicates) for α and β subunits are highlighted in light blue and light red respectively. C) The structure of NA3 glycan. Only the branched monosaccharide residues, namely α-mannose (αMan), β-*N*-acetylglucosamine (βGlcNAc) and β-galactose (β-Gal) were considered for hydrogen-bonding (H-bond) analysis in MD simulation. D) The number of H-bonds between the branched monosaccharide residues on NA3 glycan at αAsn52 and the β subunit in 150 ns MD simulation. The data were from three replicates.
